# The intersection of artificial intelligence and lung nodule research: current applications and future prospects

**DOI:** 10.1097/JS9.0000000000004595

**Published:** 2026-01-13

**Authors:** Linfeng Wang, JunHao Yu, Yue Luo, JiaYi Nie, XinYue Ge, Yue Li, BaoJin Hua, Rui Liu

**Affiliations:** Department of Oncology, Guang’anmen Hospital, China Academy of Chinese Medical Sciences, Beijing, China

**Keywords:** artificial intelligence, bibliometric analysis, deep learning, lung nodules, machine learning

## Abstract

Lung cancer represents a primary global cause of cancer-related mortality, imposing substantial healthcare burdens on both patients and public health systems. Pulmonary nodules, as early-stage manifestations of lung cancer, exhibit considerable morphological heterogeneity. Consequently, precise identification and clinical management of these nodules are critical for effective lung cancer prevention. In recent years, artificial intelligence (AI) has emerged as a transformative component in modern oncology, providing advanced tools for end-to-end pulmonary nodule management. This review systematically analyzes existing literature through bibliometric assessment to synthesize AI applications across the pulmonary nodule care continuum. AI-powered clinical decision support systems and personalized treatment planning are reshaping precision oncology paradigms. Current research advancements and prevailing challenges are critically examined to identify potential future breakthroughs. The comprehensive synthesis presented herein aims to establish a foundational conceptual framework for researchers and clinicians, while facilitating efficient translation of AI technologies into clinical practice for pulmonary nodule diagnosis and therapy.

## Introduction

Pulmonary nodules refer to round or oval-shaped shadows in the lung with a diameter of ≤3 cm as shown in imaging studies. Based on the presence of solid components, they can be classified into solid nodules and subsolid nodules (SSNs), with subsolid pulmonary nodules further divided into pure ground-glass nodules (pGGNs) and mixed ground-glass nodules^[1]^. Current evidence indicates that persistent pulmonary nodules constitute a principal early imaging indicator of lung cancer. Consequently, precise characterization and therapeutic intervention are pivotal for reducing lung cancer mortality rates^[2]^. The development and widespread implementation of low-dose spiral computed tomography (CT) screening have substantially elevated pulmonary nodule detection rates in recent years. In a large-scale epidemiological study conducted on a screening cohort of 4.8 million individuals, the prevalence of pulmonary nodules was found to be 32.7%. However, malignancy is confirmed histologically in only 4% of detected cases. This significant disparity underscores the considerable diagnostic heterogeneity inherent in pulmonary nodules and necessitates precision diagnostic strategies^[3]^. Traditional pulmonary nodule diagnosis relies on radiologists’ subjective interpretation, encountering inherent limitations including significant inter-reader variability, high missed-detection rates for subcentimeter nodules, and indeterminate benign-malignant differentiation. The continuous refinement of artificial intelligence (AI) algorithms and advances in multimodal data fusion technologies are fundamentally restructuring pulmonary nodule management paradigms. By processing vast volumes of chest CT imaging data with unprecedented efficiency, AI is transforming diagnostic approaches from experience-based to quantitatively driven frameworks^[4]^. Driven by technological innovations, AI facilitates precision therapy frameworks for pulmonary nodules. These AI-driven systems provide clinical decision support for treatment strategy optimization, thereby enhancing translational precision medicine applications in lung cancer prevention and control.

**HIGHLIGHTS**

- This is the first comprehensive exposition of the overall structure, current trends, and research hotspots in artificial intelligence for lung nodule studies.
- Utilization of advanced bibliometric methods to reveal key trends and emerging focal points.
- Publication volume has grown steadily from 2015 to 2024, accelerating markedly post-2024.
- Keyword analysis reveals the extensive use of deep and machine learning models in lung nodule detection.
- The challenges facing this research domain include data heterogeneity, diminished trust between clinicians and patients, and the absence of regulatory and ethical frameworks.

The intersection of pulmonary nodules research and AI has generated substantial literature requiring systematic synthesis to delineate technological evolution and knowledge architecture. Unlike qualitative reviews, bibliometrics quantitatively analyzes research dynamics through large-scale data, revealing latent interconnections and conceptual evolution patterns^[5]^. Despite intensified research activity at the pulmonary nodules-AI nexus, systematic bibliometric investigations remain conspicuously absent. This deficiency impedes the deconstruction of knowledge system evolution and the precise characterization of clinical translation chokepoints. To address these critical gaps, the present investigation integrates bibliometric and visualization analytics to comprehensively map the research landscape through a tripartite analytical framework: quantitative assessment of temporal trends, influential contributors, and collaborative topologies; technical evaluation of domain hotspots and developmental trajectories via keyword cooccurrence networks; and diagnostic identification of implementation bottlenecks in clinical pathways. The resultant science mapping framework establishes an empirically derived roadmap for strategic research prioritization, technological optimization pipelines, and clinical decision architecture development, thereby accelerating deployment of AI-driven precision diagnostic systems for pulmonary nodules. This work complies with the TITAN Guidelines 2025 – governing declaration and use of AI^[6]^, with the guideline checklist provided in Supplemental Digital Content Supplement 1 available at: http://links.lww.com/JS9/G526.

This paper is structured into seven methodologically organized sections. It begins with a review and critical assessment of AI applications in pulmonary nodules research. Next, the bibliometric methodology and analytical framework employed in this study. The following section presents the empirical findings derived from a comprehensive bibliometric analysis. Subsequent discussion addresses the implications of these bibliometric results, addresses challenges in AI implementation, and offers constructive recommendations. Methodological limitations and potential biases are then rigorously evaluated. Finally, the paper synthesizes substantive findings and proposes translational research directions for future studies.

## Application of artificial intelligence technology in the research field of pulmonary nodules

**Figure 1.**
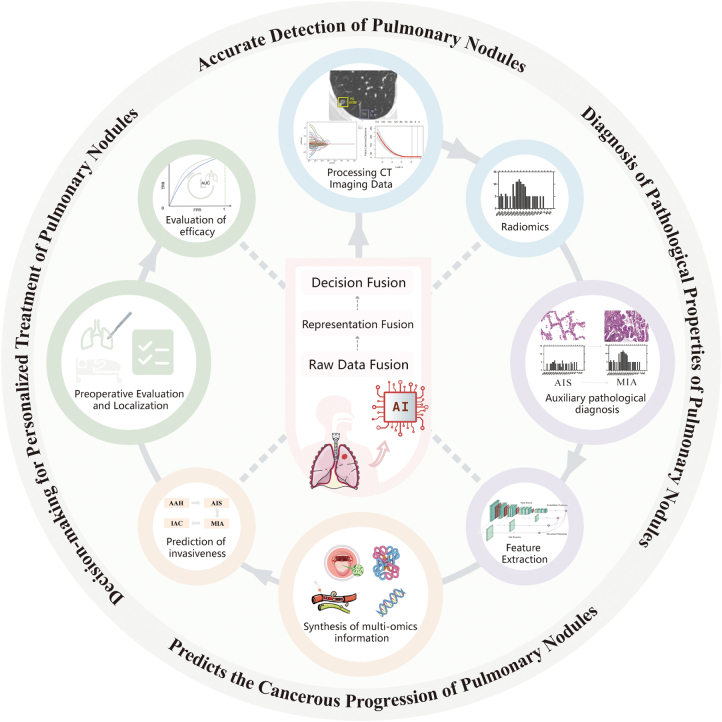
The application of artificial intelligence technology in the research field of pulmonary nodules.

AI leverages cutting-edge advances from computer science, life sciences, and clinical medicine to enable the development of systems capable of human-like cognitive tasks, including medical image analysis, pathological interpretation, and clinical decision support^[7,8]^. In recent years, there has been considerable evidence to suggest that AI technology has demonstrated tremendous application potential and wide clinical value in the full-cycle management of pulmonary nodules. The AI-driven precision diagnosis and treatment system for pulmonary nodules comprehensively encompasses core processes, ranging from initial automated detection, precise characterization, and risk assessment, to optimization decision-making of treatment plans and posttreatment prognostic evaluation. In this closed-loop diagnostic management pathway, AI systems have become indispensable intelligent decision support tools, providing physicians with robust support based on extensive data and complex algorithms, significantly enhancing the precision and efficiency of the entire process of diagnosis and treatment for pulmonary nodules (Fig. 1).

### AI-assisted precision detection of pulmonary nodules

Substantial evidence demonstrates that low-dose spiral CT screening significantly enhances early lung cancer detection and reduces mortality rates^[9,10]^. However, screening accuracy remains heavily dependent on radiologists’ interpretive expertise, inevitably leading to missed diagnoses and interpretive errors in large-scale screening programs. The integration of AI introduces transformative solutions for these inherent limitations.

Recent advancements in deep learning, particularly convolutional neural networks (CNNs), have propelled substantial progress in medical image analysis. CNNs effectively extract hierarchical image features through multilayered architectures, enabling high-precision classification and recognition tasks. In pulmonary imaging, AI algorithms precisely delineate lung fissural anatomy and differentiate nodules from surrounding parenchymal structures, consequently reducing false positive rates. Empirical studies reveal that optimized 3D U-Net integration models achieve near-expert segmentation proficiency with accelerated processing speeds, demonstrating 92% sensitivity and 82% specificity^[11]^. This technological advancement demonstrates transformative potential for improving clinical workflow efficiency. Moreover, AI-assisted pulmonary imaging systems demonstrate high capability in detecting pulmonary nodules. A retrospective multicenter cohort analysis of 12 889 patients showed that after implementing AI-assisted diagnostic systems for pulmonary nodules in two hospitals, the detection rates increased significantly – by 7.26% and 15.93%, respectively – with sustained performance improvement observed during long-term use^[12]^. In another study evaluating a deep learning-based computer-aided detection (DL-CAD) system for lung nodules detection and characterization, the DL-CAD system achieved a detection rate of 86.2%, significantly outperforming radiologists, whose detection rate was 79.2%^[13]^. These results affirm the potential of AI to enhance diagnostic accuracy and expedite radiological interpretation, thereby offering clinicians more efficient and precise diagnostic support.

Contemporary research extensively evaluates deep learning architectures for pulmonary nodule detection, with foundational algorithms encompassing U-Net variants, region proposal networks, and residual networks^[14]^. A pioneering study has combined motion vector CNNs with adaptive enhancement algorithms to construct a new nodule recognition framework. This model has been demonstrated to exhibit excellent performance with regard to feature extraction and computational scalability. The validation results on the Early Lung Cancer Action Plan dataset demonstrate an accuracy rate of 99%, sensitivity of 100%, and specificity of 98%, thus substantiating the methodology’s efficacy in terms of diagnostic precision^[15]^. Another study introduced the NC-UNet model, which utilizes a weakly labeled strategy to diminish the need for meticulously annotated data. The model incorporates a robust preprocessing pipeline – including threshold segmentation, morphological operations, and region of interest (ROI) standardization – to effectively suppress background tissue interference. Its principal innovation involves the integration of corner detection mechanisms within the U-Net framework, facilitating efficient use of weakly labeled datasets and accurate localization of candidate nodules, substantially improving lung nodules detection in CT imaging. On the publicly available LIDC-IDRI dataset (n = 814), the model achieved an area under the curve (AUC) of 0.938 for pulmonary nodule localization. Furthermore, internal validation using an independent cohort (n = 822) achieved an AUC of 0.943, corroborating the model’s high diagnostic accuracy and promising potential for clinical application^[16]^. Furthermore, a lung nodule detection model was developed employing a hybrid strategy that integrates convolutional and long short-term memory neural networks. Trained on 24 107 real-world patient cases, the model incorporates multimodal features from clinical text and CT images, achieving AUC values of 0.910 and 0.887 on internal and external test sets, respectively, which demonstrates excellent recognition performance and promising potential for clinical translation^[17]^.

### AI enhancing diagnostic efficacy for differentiating benign and malignant pulmonary nodules

Once lung nodules have been detected, radiologists need to extract and analyze their key features, such as size, morphological characteristics, and density distribution, in order to assess the risk of malignancy. However, traditional analysis methods are significantly limited when it comes to imaging biomarkers, particularly complex morphological patterns and texture heterogeneity, both of which are critical for characterizing tumors. Although these nodule attributes are key to malignant risk classification, traditional methods are relatively poor at capturing subtle features. In contrast, AI systems demonstrate a greater ability to discriminate between such complex features. A large-scale, multicenter validation study (N = 23 336) demonstrated that AI significantly outperformed human radiologists in detecting malignant pulmonary nodules, with an accuracy of 97.2% compared to 86.4%, representing an absolute performance improvement of 10.8%^[18]^. Recent research has proposed an automated system based on a multiscale residual network (MResNet) architecture for integrated lung nodules feature extraction and benign/malignant classification. This innovative model demonstrates exceptional diagnostic performance (accuracy: 99.1%; sensitivity: 98.6%; specificity: 97.9%). Thanks to its superior feature representation capabilities, the system has the potential to reduce the need for unnecessary invasive examinations by up to 38%^[19]^. A separate study developed a deep learning-based diagnostic system that demonstrated robust detection performance. On an external test set comprising 100 scans from 100 patients, the AI system achieved sensitivities of 94.3% for benign nodules, 96.9% for primary lung cancer, and 92.0% for metastatic lesions. These sensitivity levels were comparable to or higher than those of radiologists, offering enhanced potential for early diagnosis of pulmonary nodules^[20]^. In addition to CT image analysis, AI-integrated, shape-aware, robot-assisted bronchoscopy has emerged as an innovative diagnostic technique. Recent multicenter trials have validated its efficiency and safety in malignant nodule sampling, with a sensitivity of 90.8% and a negative predictive value of 90.0%. This offers a minimally invasive alternative to traditional biopsy techniques^[21]^. The AI-assisted bronchoscopic robot system developed by Zhang *et al*^[22]^ demonstrated significant advantages in experimental evaluations. *In vitro* experiments showed that the operational error of novice physicians assisted by AI (17.63 ± 0.46 pixels) was significantly lower than that of expert operators (31.45 ± 1.19 pixels). *In vivo* experiments further revealed that the AI-assisted group achieved a lower operational error (11.38 ± 0.16 pixels) compared to the expert group (16.26 ± 0.27 pixels), along with a reduced number of required interventions. This study indicates that AI-assisted technology holds promise for enhancing the accuracy and efficiency of bronchoscopic procedures, with important potential for improving the diagnosis of pulmonary nodules and other lung diseases^[22]^.

AI demonstrates dual capabilities in pulmonary nodule assessment: precise characterization of internal nodular features and robust integration of multisource data. Multiomics diagnostic frameworks that combine liquid biopsies, genomic profiles, and AI-powered imaging analysis offer unprecedented potential for early detection and precision management of pulmonary nodules. This approach effectively addresses the well-documented limitations of single-biomarker methodologies in pulmonary nodule screening, which frequently exhibit insufficient sensitivity and low specificity. Current research paradigms consequently prioritize the integration of liquid biopsies and AI technologies into diagnostic pathways to enhance early lung cancer identification accuracy. A study incorporating data from 1380 participants confirmed that the PulmoSeek Plus model – integrating imaging information, demographic and clinical characteristics, and circulating tumor DNA (ctDNA) methylation – exhibits excellent discriminatory performance. Using a two-threshold strategy (0.65 and 0.89), it could reduce unnecessary surgeries by 89% and delayed treatments by 73%, indicating substantial potential for clinical translation^[23]^. A further study demonstrated that a data-driven lung nodule malignancy risk assessment system (C-Lung-RADS) integrating imaging information, demographic characteristics, and follow-up data exhibits superior performance. In both the internal and external validation datasets, the multidimensional system exhibited significant advantages over the conventional Lung-RADS system. The internal validation results demonstrated that the sensitivity of C-Lung-RADS was 20% higher than that of the Lung-RADS system. The external validation process further substantiated the performance advantages of the C-Lung-RADS system, with the system demonstrating a sensitivity of 87.1%, which is significantly higher than the 63.3% sensitivity achieved by Lung-RADS. The findings unequivocally substantiate the hypothesis that AI-based methodologies for predicting benignity or malignancy employing multimodal data exhibit a pronounced technical superiority over conventional single-dimensional approaches^[24]^. Another study involving 501 patients with ground-glass nodules (GGNs) demonstrated that a multimodal model integrating clinical features, biomarkers (TPI-1 and miR-206), and deep radiomics could accurately distinguish benign from malignant GGNs, achieving an AUC of 0.9^[25]^. The fusion of multimodal biomarkers and AI provides a new perspective and pathway for the early diagnosis of pulmonary nodules, and future research will further promote the development of this field, laying a more solid foundation for early lung cancer screening and personalized treatment.

Moreover, AI-assisted, precise benign and malignant diagnosis significantly optimizes the follow-up management costs of pulmonary nodules. Integrating information from AI-driven high-precision nodule risk assessment models allows for more accurate clinical risk stratification. This strategy effectively identifies low-risk nodules, significantly reducing the frequency of unnecessary follow-up examinations, thereby saving considerable costs for the healthcare system^[26]^.

### Artificial intelligence prediction of lung nodules invasiveness

In 2021, the World Health Organization published a histological classification of lung tumors that categorizes lung adenocarcinoma into adenomatous precursor lesions, minimally invasive adenocarcinoma (MIA), and invasive adenocarcinoma (IAC). Among these, adenomatous precursor lesions encompass atypical adenomatous hyperplasia (AAH) and adenocarcinoma *in situ* (AIS)^[27]^. Persistent pulmonary nodules may manifest as one of several distinct pathological entities^[2]^. Given the substantial divergence in therapeutic protocols and clinical outcomes across varying stages of lung adenocarcinoma invasion, precise histopathological staging and invasiveness assessment provide critical decision-making guidance for personalized management strategies.

AI technology can transform medical imaging into quantifiable high-dimensional data, extracting multidimensional quantitative information that includes nodule size, morphological features, and tumor spatiotemporal heterogeneity, thereby deeply exploring the intrinsic association between these features and lung nodules progression. Research has shown that AI-based models exhibit high sensitivity and specificity in predicting the invasive subtypes of early lung adenocarcinoma, highlighting their potential in guiding personalized treatment, making noninvasive classification of lung adenocarcinoma subtypes possible. Specifically, AI models can effectively differentiate AIS, MIA, and IAC by analyzing the mean density, volume, malignancy probability score, and morphological signs such as spiculation and pleural indentation in CT images of nodules. A retrospective study (distinguishing AIS/MIA/IAC) of 223 early-stage lung adenocarcinoma cases presenting as pulmonary nodules was conducted to evaluate the predictive value of imaging characteristics combined with an AI system for invasive subtypes. The AI-assisted diagnostic system exhibited robust performance in distinguishing invasive subtypes, with a sensitivity of 81.76%, specificity of 92.45%, and an AUC of 0.871 in binary classification. In the three-category classification task, it attained an accuracy of 83.86% and an AUC of 0.879. These findings indicate that CT image-based AI tools have the potential to serve as an auxiliary means for predicting the invasiveness of lung adenocarcinoma, thereby providing valuable support for clinical diagnosis and treatment decision-making^[28]^. Furthermore, a retrospective study on pGGN developed a fully automated deep learning model, Lung-PNet. This model differentiates preinvasive lesions (AAH/AIS) from IAC by automatically analyzing deep imaging features. It demonstrates significantly superior diagnostic performance in large-scale dataset validation compared to traditional imaging analysis methods and previous models. The model provides an efficient and objective auxiliary tool for noninvasive assessment of pGGN characteristics and clinical decision-making^[29]^.

GGNs are typically characterized by indolent progression but carry a risk of transitioning to a rapid progression phase^[30]^. Early identification of nodules at risk prior to rapid progression is critical for formulating optimal diagnosis and treatment strategies. AI has been extensively employed in research predicting GGNs growth. This is achieved by converting medical images into quantifiable high-dimensional data, thereby extracting multidimensional information encompassing nodule size, morphological characteristics, and spatio-temporal heterogeneity. This facilitates a deeper analysis of their correlation mechanisms with nodule progression. A study analyzing 2523 cases of SSNs with at least 2 years of follow-up developed and compared a deep learning model and a radiomics model for predicting nodule growth. The deep learning model significantly outperformed the radiomics model in both the validation cohort (AUC = 0.858) and the external test cohort (AUC = 0.862). Notably, using only baseline CT images, the deep learning model achieved AUCs of 0.855 and 0.821 in the NLST validation and external test sets, respectively, demonstrating its strong potential for predicting SSN progression^[31]^. Another study used a three-dimensional CNN to automatically identify and match GGNs in multitime-point CT images, eliminating the need for image registration. This model was integrated into a fully automated workflow encompassing nodule detection, matching, and growth prediction, achieving an overall sensitivity of 92.0% and an accuracy rate of 88.4%^[32]^. In another retrospective study involving 286 patients (419 pGGNs), a deep learning model was developed to predict the growth of pGGNs. The model demonstrated strong predictive performance, achieving an AUC of 0.79 in the internal validation cohort and 0.70 in the external validation cohort^[33]^. Furthermore, a clinical study employed a language model-assisted system combined with longitudinal CT images to automatically evaluate the dynamic progression of GGNs. This system identifies subtle changes in nodule size, density, and morphology, and quantifies their malignant risk and growth trajectory. By continuously monitoring key imaging features such as calcification patterns and marginal sharpness, the model provides an objective basis for formulating personalized follow-up plans and early intervention strategies^[34]^. Notably, AI-based multiomics analysis is identifying key biomarkers associated with the transformation of indolent pulmonary nodules into invasive phenotypes. Studies indicate that the status of specific driver gene mutations is being linked to distinct radiomic features, such as tumor diameter and CT attenuation values. Furthermore, specific genomic mutation profiles within nodules are strongly correlated with invasiveness, offering promise for noninvasive early prediction of aggressive transformation^[35–37]^.

### AI-assisted personalized treatment decision support for lung nodules

**Figure 2.**
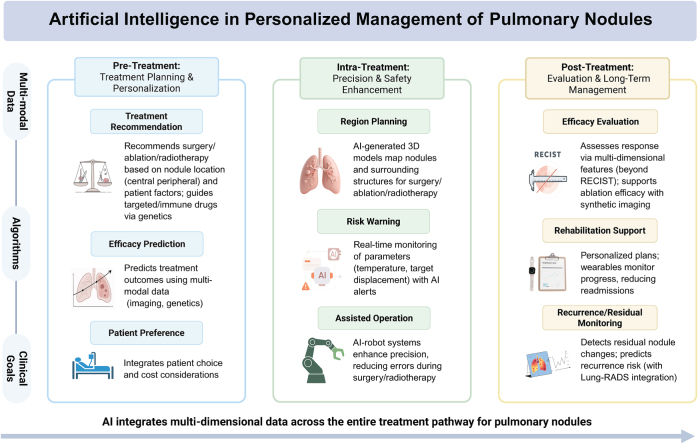
Artificial intelligence-assisted personalized treatment decision support for pulmonary nodules.

AI has become increasingly central to personalized decision support for lung nodules management, spanning the entire continuum from pretreatment assessment and treatment planning to posttreatment prognosis (Fig. 2). The capacity of deep learning models to synthesize high-resolution CT imaging, clinical history, gene expression profiles, and longitudinal follow-up data to create personalized malignant risk stratification systems is well documented. The models are predicated on predicted risk probabilities and thus provide critical diagnostic and therapeutic guidance. Imaging surveillance is recommended for low-risk nodules, in order to prevent overtreatment, while biopsy or surgery is indicated for intermediate-to-high-risk cases. Furthermore, the models recommend specific treatment modalities, such as stereotactic body radiotherapy, sublobar resection, or ablation therapy, which are tailored to the pathological subtypes of diagnosed early-stage lung cancer^[38]^. This data-driven, closed-loop decision support significantly enhances the efficacy of early lung cancer screening and treatment, facilitating the shift from population-based screening to personalized intervention in lung cancer control.

In the pretreatment evaluation of lung nodules, AI systems integrate multimodal data through deep learning neural networks, significantly enhancing the accuracy of distinguishing benign from malignant pulmonary nodules and enabling precise prediction of pathological subtypes. This provides critical support for the precise diagnosis and classification of lung cancer. By leveraging these capabilities, AI synthesizes multiple clinical variables, including pulmonary function, lesion location, and patient age, to generate objective treatment recommendations, thereby further optimizing individualized therapeutic strategies. Given the high heterogeneity of pulmonary nodules, the multidisciplinary team (MDT) approach is particularly crucial. Research demonstrates that AI also plays an important role in MDT. In a study involving 1000 Chinese lung cancer patients, the treatment plans recommended by the AI system achieved 93% consistency with MDT consensus, while reducing the average decision-making time from 120 minutes to 12 minutes, markedly improving diagnostic and treatment efficiency^[39]^. Furthermore, research indicates that EGFR is a common driver gene mutation in pulmonary nodules, whilst KRAS mutations are significantly associated with nodule aggressiveness, suggesting they may exert stage-specific regulatory roles in tumor progression^[40,41]^. Notably, the ALK gene exhibits potent oncogenic potential, capable of independently driving rapid progression of pulmonary nodules to highly invasive phenotypes even in the absence of other genetic alterations. Its clinical-pathological characteristics demonstrate a leapfrog evolution pattern from “precancerous lesions” to “invasive carcinoma.” In contrast, genes such as BRAF and HER2 possess relatively weaker driver capabilities, often resulting in tumors exhibiting indolent growth patterns when present alone^[36]^. These findings indicate that the molecular characteristics of early-stage lung cancer hold significant implications for guiding the developmental trajectory of pulmonary nodules and subsequent therapeutic approaches. However, the small size of nodules poses substantial challenges for preoperative molecular typing, as conventional needle biopsy struggles to yield sufficient tissue samples. Although novel therapies such as targeted drugs and immune checkpoint inhibitors have been explored for treating pulmonary nodules, their efficacy remains suboptimal^[42,43]^. In this context, AI technologies – particularly machine learning and deep learning – provide an innovative pathway toward “virtual biopsy” by decoding the rich biological information embedded within conventional medical images. The principal advantage of this approach is twofold: it leverages radiomics to quantitatively extract numerous subtle features from CT scans that are invisible to the human eye, and utilizes CNNs for end-to-end automated feature learning. This facilitates the development of high-precision models that correlate imaging patterns with critical molecular markers, including EGFR, KRAS, and ALK mutations, as well as PD-L1 expression levels. Such a technological advance allows clinicians to noninvasively obtain molecular profiles prior to treatment, enabling better patient stratification for targeted and immunotherapies while substantially streamlining diagnostic workflows. It demonstrates broad clinical application prospects. Empirical studies validate AI’s predictive capabilities: robust efficacy prediction models have been constructed by integrating radiomics features with clinical parameters through multimodal approaches. Studies have confirmed that AI can develop robust predictive models for treatment efficacy by integrating multimodal radiomic features and clinical parameters. Such models demonstrate exceptional performance in predicting treatment response and survival outcomes for lung cancer patients receiving immune checkpoint inhibitors and EGFR-targeted therapies^[44,45]^. Crucially, AI systems incorporate not only clinical parameters but also patient-centered factors, including treatment preferences and economic considerations. Through dynamically adjusted algorithms, these systems synthesize multidimensional information to formulate clinically effective treatment strategies aligned with patient needs^[46,47]^.

In the context of surgical interventions, thermal ablation, and stereotactic body radiotherapy for pulmonary nodules, AI-facilitated real-time monitoring systems achieve unprecedented procedural precision through synergistic integration of multimodal sensing technologies, including electromagnetic positioning, intraoperative ultrasonography, and infrared thermography, coupled with advanced dynamic analytics. These systems enable continuous quantification of critical intraoperative parameters such as thermal ablation field distributions, radiotherapy target displacement vectors, and real-time instrument proximity to critical vasculature and bronchial structures. This paradigm significantly elevates both patient selection criteria accuracy and treatment area delineation precision. Concurrently, AI-driven robotic platforms autonomously reconstruct high-resolution three-dimensional pulmonary models from preoperative CT datasets, providing intuitive visualization of spatial relationships between target nodules and adjacent vital anatomical structures, including vascular networks and tracheobronchial architecture. Empirical validation confirms AI algorithms convert standard two-dimensional imaging into interactive 3D visualizations, elevating anatomical identification accuracy to 0.87 – representing an 11.5% improvement over conventional methods – while simultaneously reducing diagnostic misjudgments by 41% and compressing preoperative planning timelines by 25%. Further enhancing clinical pathways, AI-enhanced robotic navigation synthesizes nodule topography, pulmonary functional metrics, and comorbidity profiles to construct machine learning-based decision trees that optimize surgical approach selection – achieving 0.85 accuracy with 35% error reduction – while precision-mapping resection pathways and instrument trajectories. These integrated capabilities collectively redefine precision thresholds in thoracic oncology interventions^[48]^. In radiotherapy or ablation procedures, AI supports automatic delineation of target areas and real-time simulation of dose distribution, dynamically adjusting energy delivery based on imaging feedback to minimize damage to surrounding tissues^[49]^. During intraoperative procedures, the use of high-precision robotic arm motion control and reinforcement learning decision models can effectively overcome physiological tremors and visual errors associated with traditional operations^[50]^. Clinical studies have demonstrated that the AI-robotic-assisted system enhances intraoperative target localization accuracy, achieves a 100% success rate in puncture procedures, reduces operative time, and thereby establishes an innovative paradigm for minimally invasive precision therapy^[51]^. Additionally, intelligent feedback systems, supported by CNNs, construct real-time risk warning models that provide quantifiable operational suggestions through multimodal monitoring data, significantly enhancing operational safety and controllability^[52]^.

AI also demonstrates significant value in the posttreatment follow-up care of patients with lung nodules. In terms of efficacy evaluation, AI models can quantify the morphological characteristics of nodules and the dynamic changes in the tumor microenvironment before and after treatment through CT imaging, overcoming the limitations of the traditional RECIST standard that relies on tumor diameter, accurately identifying complex response patterns from different treatments. For example, breakthroughs based on generative adversarial networks (GANs) have achieved the direct synthesis of high-resolution pseudo-contrast-enhanced ultrasound (pseudo-CEUS) images from ultrasound, providing a noninvasive assessment method for ablation efficacy^[53]^. In prognostic stratification, AI synthesizes preoperative CT characteristics, longitudinal follow-up data, and high-risk indicators (including tumor topography and growth kinetics) to develop personalized recurrence risk models. These models accurately predict critical outcomes – such as survival trajectories and disease progression timelines – by incorporating multidimensional clinical variables: patient age, tumor staging, genetic mutation profiles (notably EGFR status), and PET-CT metabolic signatures^[54]^. Such genomic and molecular characterization proves indispensable for guiding subsequent therapeutic decisions and prognostic evaluations.

Notably, AI-driven approaches revolutionize mutation detection through computational histopathology: weakly supervised multiinstance learning models applied to postoperative H&E slides demonstrate robust performance in predicting EGFR mutations. This technique achieves diagnostic reliability comparable to molecular assays while substantially reducing tissue consumption, turnaround time, and testing costs – a breakthrough validated across both internal cohorts and diverse external datasets^[55]^. Recent research has overcome the limitations of conventional genomic analysis by combining imaging-based features of the tumor immune microenvironment and heterogeneity with clinical and pathological indicators. This has successfully established an innovative predictive model for the prognosis of patients undergoing surgery for nonsmall cell lung cancer. The multimodal fusion approach reveals spatial correlations between imaging phenotypes and the immune microenvironment, providing new insights for prognostic assessment^[56]^. Complementing this, deep learning frameworks perform risk stratification by extracting prognostic features from postradiotherapy CT scans. These models utilize saliency mapping to visually delineate the contribution of specific intratumoral and peritumoral regions to prognostic outcomes, offering unprecedented biological interpretability^[57]^.

The management of residual nodules following surgical resection presents ongoing clinical challenges, underscored by evidence indicating that approximately 40.2% of GGNs demonstrate progression within 3 years postprimary lesion removal^[58]^. AI systems address this complexity through automated detection and quantitative tracking of morphological evolution in subtle residual lesions, including volumetric expansion and density variations. By integrating these metrics with dynamic risk-prediction algorithms, AI proactively identifies transition markers from indolent to aggressive phenotypes, thereby informing evidence-based decisions regarding tailored therapeutic interventions. In optimizing pulmonary nodule surveillance strategies, AI algorithms leverage serial imaging data to quantify doubling time kinetics and morphological evolution, establishing synergistic integration with the Lung-RADS classification framework. This approach enables risk reclassification for 30% of baseline Lung-RADS 3 cases – permitting extended 12-month monitoring intervals – while identifying clinically significant subsets (0.2%) among underclassified Lung-RADS 1–2 nodules requiring intensified surveillance^[26]^. By reducing unnecessary interventions and optimizing healthcare resource allocation, this integrated methodology establishes a risk-stratification-adaptive monitoring-resource optimization management pathway.

In the application of AI to the holistic management of pulmonary nodules, although it demonstrates considerable potential in nodule detection, malignancy risk assessment, and invasiveness prediction, it continues to face the challenge of a high false positive rate, which has become a major bottleneck hindering its widespread clinical adoption. This issue is primarily reflected in the tendency of AI systems to misclassify benign lesions as high-risk malignancies, leading to unnecessary follow-up examinations, invasive procedures, and patient anxiety, thereby significantly compromising the precision and cost-effectiveness of lung cancer screening. The risk of misjudgment is especially prominent in complex cases. For instance, certain indolent adenocarcinomas (such as AIS) and benign inflammatory conditions may both present as GGNs on imaging, with highly overlapping features. However, AI exhibits relatively low sensitivity in detecting GGNs – at the highest sensitivity setting, it can identify only up to 50% of such nodules – resulting in reduced diagnostic accuracy^[59–61]^. Furthermore, while features such as lobulation and spiculation are critical indicators used by AI to determine malignancy, some benign nodules also exhibit these characteristics. Inflammatory pseudotumors displaying lobulation or spiculation, for example, may increase the rate of AI misclassification^[62,63]^. In addition, the missed detection rate of AI rises significantly for small nodules or those in challenging locations (e.g., near the pleura), potentially delaying treatment^[64]^. Moreover, the accuracy of AI algorithms still requires improvement compared to traditional manual classification of pulmonary nodules. In the LUNGx Challenge, the AI group achieved lower AUC values than the radiologist group (0.50–0.68 vs. 0.70–0.85), indicating that AI technology has not yet reached a level where it can replace human expertise in classifying pulmonary nodules^[65]^.

The persistently high false positive rate can be attributed to three main factors:

First, at the data level, issues such as class imbalance in training sets, variations in imaging equipment and parameters, inconsistencies in annotation, and insufficient sample diversity lead models to adopt conservative diagnostic approaches with limited generalizability. Second, at the algorithmic level, inherent biases in feature extraction cause models to overemphasize local imaging patterns while neglecting broader clinical context. They also exhibit limited ability to distinguish complex benign conditions such as inflammation and granulomas. Third, at the clinical validation level, the lack of large-scale multicenter prospective studies means that model performance often declines significantly when transitioning from experimental settings to real-world clinical environments. To systematically address these challenges, multifaceted strategies are required: Technologically, federated learning frameworks can be employed to integrate multicenter heterogeneous data while preserving privacy, thereby improving model generalizability. Meanwhile, multimodal fusion models should be developed to combine imaging features with clinical information (e.g., age, smoking history, tumor markers) for a more comprehensive risk assessment system. In terms of model interpretability, attention mechanisms and feature visualization techniques should be incorporated to clearly illustrate decision-making processes and enhance clinical trust. From a clinical integration perspective, large-scale prospective trials should be conducted to validate AI efficacy in real-world settings. Moreover, AI should be seamlessly embedded into radiology information systems (RIS) to optimize human-AI collaborative diagnostic workflows. Only through dual advancement in both technological innovation and clinical practice can we establish an efficient and reliable intelligent diagnostic paradigm for pulmonary nodules.

In summary, AI-driven systems deliver real-time clinical decision support through sophisticated integration of multimodal imaging and patient-specific data, optimizing individualized therapeutic applications. The emerging paradigm of collaborative intelligence – where AI augments clinical expertise – demonstrates transformative potential in pulmonary nodule management. As technological maturation converges with enhanced model interpretability, AI is poised to assume increasingly pivotal roles within clinical workflows, advancing both therapeutic outcomes and population health metrics. The inevitable evolution toward clinician-AI symbiosis will establish intelligent clinical decision support systems as fundamental components of modern healthcare infrastructure, directly enabling precision medicine implementation. Future progress necessitates multidimensional bibliometric analysis to systematically map AI-nodule research integration trajectories, delineate technological evolution patterns, identify clinical translation barriers, and elucidate breakthrough pathways for next-generation diagnostics.

## Materials and methods

### Retrieval strategy

This study employs a systematic literature review method, conducting search queries on the Web of Science (WoS) Core Collection with a time limit from January 2015 to December 2024, including types: articles and reviews, search query: (((TS=(“Artificial Intelligence” OR “Machine Intelligence” OR “Computational Intelligence” OR “Computer Reasoning” OR “Deep Learning” OR “Hierarchical Learning” OR “Machine Learning” OR “Federated Learning” OR “Transfer Learning” OR “Natural Language Processing” OR “Large Language Model” OR “Radiomic” OR “Convolutional Neural Network*” OR “Visual Geometry Group Network*” OR “Residual Neural Network” OR “Efficientnet” OR “Densely Connected Convolutional Network*” OR “Computer-Assisted Diagnosis” OR “Computer-Aided Diagnosis”)) AND TS=(“Pulmonary Nodule*” OR “Lung Nodule*” OR “Coin Lesion*” OR “Solitary Ground Glass Opacity” OR “Subsolid pulmonary nodule*” OR “Ground-glass nodule*” OR “subsolid nodule*”)) AND DOP=(2015-01-01/2024-12-31) AND DT=(Article OR Review)) AND LA=(English).

### Research selection and data extraction

Two researchers (LFW, JHY) independently screened the titles and abstracts of the literature, excluding studies that did not meet the inclusion criteria. In case of disagreement during the final inclusion phase, a third reviewer (NJY) made the judgment to resolve it. The total citation counts, number of publications, WoS categories and subjects, journals, countries, keywords, authors, and affiliated institutions of related articles were obtained from the WoS database.

### Data analysis

The final collection of literature was loaded in pure text format into CiteSpace 6.3^[66]^, VOS viewer 1.6.20^[67]^, and the R package bibliometrix by the two researchers for bibliometric analysis^[68]^. The quantity of literature, authors, publication years, countries/regions, institutions, keywords, and references were evaluated. The 2024 Journal Citation Reports (JCR) were used to retrieve journal rankings and impact factors.

## Results

### Search process

**Figure 3.**
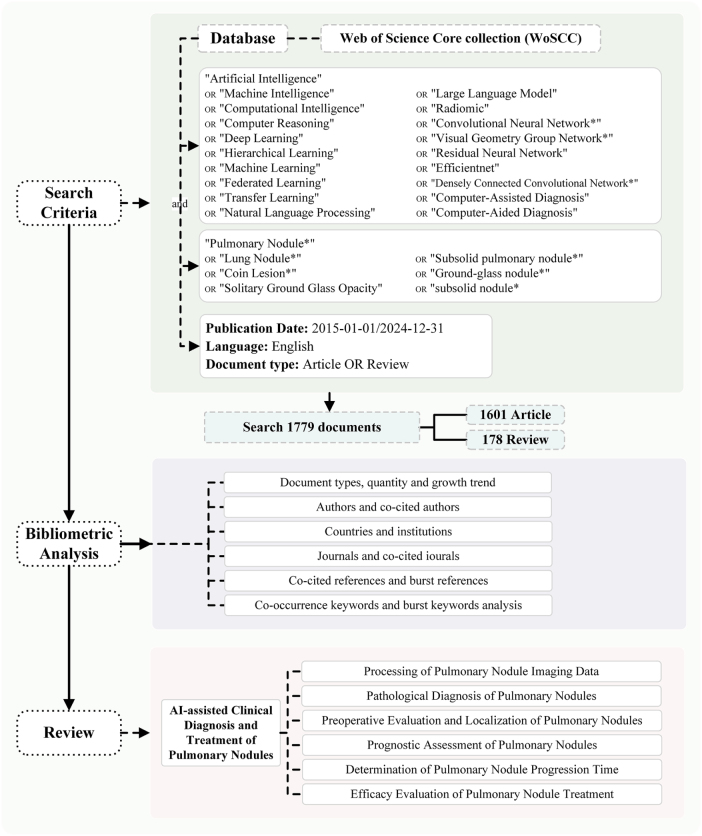
Protocol details of this study.

The research strategy identified 1908 records in the WoS database. After screening titles and abstracts, 129 records were excluded, which included conference abstracts (n = 100), early access (n = 22), editorial materials (n = 16), retracted publications (n = 13), corrections (n = 9), thesis reports (n = 6), letters (n = 3), book chapters (n = 2), and data papers (n = 1), as they were not directly related to this study. Ultimately, 1779 articles met the inclusion criteria and were included in this bibliometric analysis (Fig. 3).

### Analysis of the number of publications

**Figure 4.**
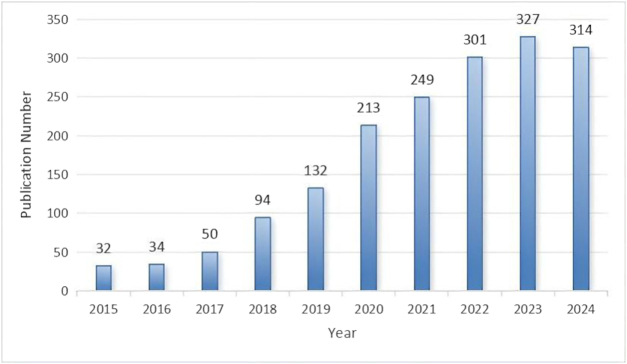
Annual publications and growth trend of artificial intelligence and pulmonary nodules research from 2015 to 2024.

The number of publications serves as a clear indicator of research trends within a specific field over a given period. Figure 4 illustrates the annual publication trend in AI research on pulmonary nodules. A total of 1779 papers were retrieved from 2015 to 2024. Over the past decade, the number of related publications showed a gradual increase, with a particularly rapid surge occurring between 2019 and 2024, during which 1536 papers (86.3%) were published. The year 2023 marked the peak with 327 publications, after which the number began to stabilize.

### Analysis of the contributions of countries

**Figure 5.**
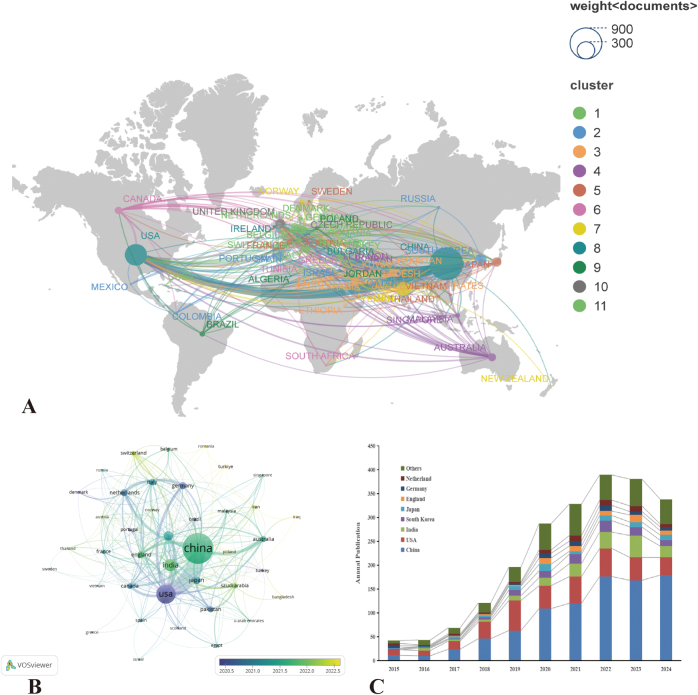
(A) world map showing the distribution of global publications on AI in pulmonary nodules; (B) overlay visualization map; (C) bar graph of the top eight productive countries/regions.

Bibliometric analysis has documented contributions from 77 countries/regions to the AI-based management of lung nodules. Figure 5 synthesizes national publication metrics and collaboration networks, where node size in Figure 5A–B corresponds to publication frequency and connecting line thickness denotes collaboration intensity, with Figure 5C delineating annual output trajectories. China dominates with 899 publications (50.5% of global output), followed by the United States (386; 21.7%) and India (171; 9.6%). Demonstrating remarkable acceleration, China’s annual output surged from 11 publications in 2015 to 179 in 2024, while maintaining robust partnerships – most significantly with the United States, alongside South Korea, the United Kingdom, Italy, and the Netherlands. Temporal clustering analysis (Fig. 5B–C) reveals distinct national chronologies: the United States emerges as an early research pioneer (blue cluster), whereas China’s peak productivity concentrated during May 2021–early 2022 (green cluster), indicating divergent innovation cycles across global research consortia.

### Analysis of the contributions by institutions

**Figure 6.**
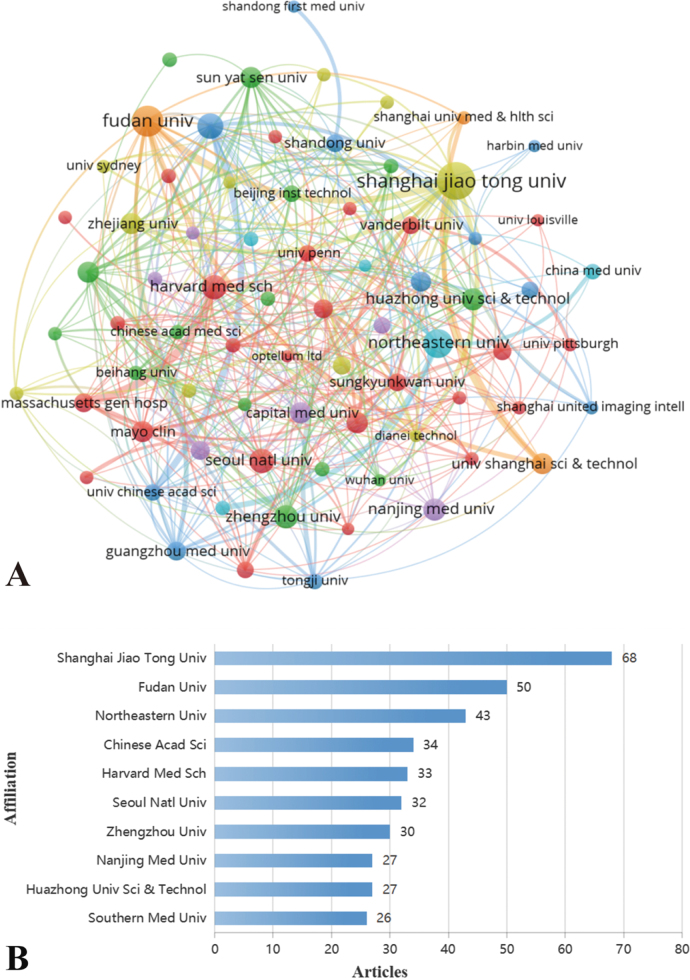
(A) A network visualization map showing the distribution of global institutions in artificial intelligence publications on lung nodules. (B) The top 10 global organizations focused on artificial intelligence.

Bibliometric analysis identified 2408 institutions that have contributed to academic publications in this field; the corresponding publication trends are visualized in Figure 6. As shown in Figure 6A, the top five contributing institutions were Shanghai Jiao Tong University (68 publications), Fudan University (50 publications), Northeastern University (43 publications), the Chinese Academy of Sciences (34 publications), and Harvard Medical School (33 publications). Together, these institutions produced 228 papers – a number substantially higher than that of other institutions. Notably, four of the top five institutions are based in China, with Harvard Medical School being the only representative from the United States. The collaboration network, illustrated in Figure 6B, divided 204 institutions into six distinct clusters, with the dominant red cluster comprising 25 institutions. Harvard Medical School served as a central hub within the network, demonstrating extensive collaborative ties across multiple research alliances.

### Analysis of author impact and collaboration

A total of 8587 authors participated in the research on AI and lung nodules. Table 1 lists the top 10 authors with the most published articles related to the association between lung nodules and AI, along with their total number of articles and H-index. Among them, the most active authors are Jin Mo Goo and Colin Jacobs, each having published 18 relevant studies, with H-indices of 60 and 31, respectively. Following them is Rozemarijn Vliegenthart, who published 16 related articles. Interestingly, there is no significant correlation between the number of articles and the number of citations; Robert J. Gillies published 13 related articles but received the most citations (count = 1933), surpassing other authors.

**Table 1:** The total number of articles by the top 10 authors, with the proportion and the h-indices.

Rank	Author	Articles	H-index
1	Goo, Jin Mo	18	60
2	Jacobs, Colin	18	31
3	Vliegenthart, Rozemarijn	16	51
4	Park, Chang Min	15	54
5	Qi, Shouliang	15	30
6	Qian, Wei	15	99
7	Van Ginneken, Bram	15	87
8	Gillies, Robert J.	13	107
9	Li, Weimin	13	58
10	Prokop, Mathias	13	76

**Figure 7.**
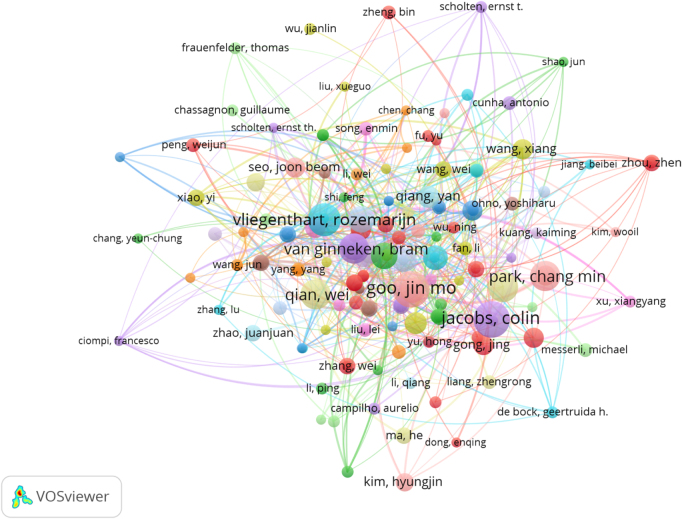
The visualization of authors in the field of artificial intelligence and lung nodule research.

Figure 7 shows the coauthorship network among the researchers, revealing their collaborative relationships. The size of the points indicates the number of publications by each author, while the color reflects different groups of authors with varying collaboration intensity. It can be seen that these groups are dispersed, with little collaboration, and do not form a large community.

In Supplemental Digital Content Supplementary Figure 1 available at: http://links.lww.com/JS9/G526, the coauthorship network of cocited authors is shown, where the size of each point represents the number of publications by each author, and the color reflects different groups of authors with varying collaboration intensities, mainly divided into three main groups. Among them, Samuel G. Armato is the most frequently cited author, with a total of 765 citations, followed by Arnaud A. A. Setio (598 citations) and Denise R. Aberle (490 citations). Supplemental Digital Content Supplementary Table 1, available at: http://links.lww.com/JS9/G526 displays the top 10 cocited authors and their citation counts.

### Cocitation journal analysis and publication journal analysis

Table 2 delineates the top 10 journals ranked by cocitation frequency and publication volume in AI for lung nodules research. The foremost cocited journals include Radiology (3659 citations), Medical Physics (2306), IEEE Transactions on Medical Imaging (2287), Medical Image Analysis (1782), and European Radiology (1756) – all classified as Q1 journals in the JCRs. Regarding publication output, IEEE Access leads with 66 articles, closely followed by European Radiology (59 publications). Among the top 10 journals, five hold Q1 rankings while the remaining maintain Q2 status, establishing dual excellence in citation impact and scholarly productivity within this domain.

**Table 2:** The numbers of the top 10 journals and cocited journals in the field of artificial intelligence and lung nodule research.

Cocited journals	Quantity	JCR	IF	Published journals	Quantity	JCR	IF
Radiology	3659	Q1	15.2	IEEE Access	66	Q2	3.6
Med Phys	2306	Q1	3.2	Eur Radiol	59	Q1	4.7
IEEE T Med Imaging	2287	Q1	9.8	Front Oncol	56	Q2	3.3
Med Image Anal	1782	Q1	11.8	Med Phys	49	Q1	3.2
Eur Radiol	1756	Q1	4.7	Sci Rep	48	Q1	3.9
Lect Notes Comput Sc	1683	-	-	Diagnostics	39	Q1	3.3
Proc CVPR IEEE	1654	-	-	Quant Imaging Med Surg	39	Q2	2.3
Sci Rep	1152	Q1	3.9	Biomed Signal Process Control	34	Q2	4.9
J Thorac Oncol	1117	Q1	20.8	Cancers	34	Q2	4.4
New Engl J Med	1117	Q1	96.3	Journal of Digital Imaging	32	Q1	3.8

### Reference analysis

#### Reference cocitation analysis

**Figure 8.**
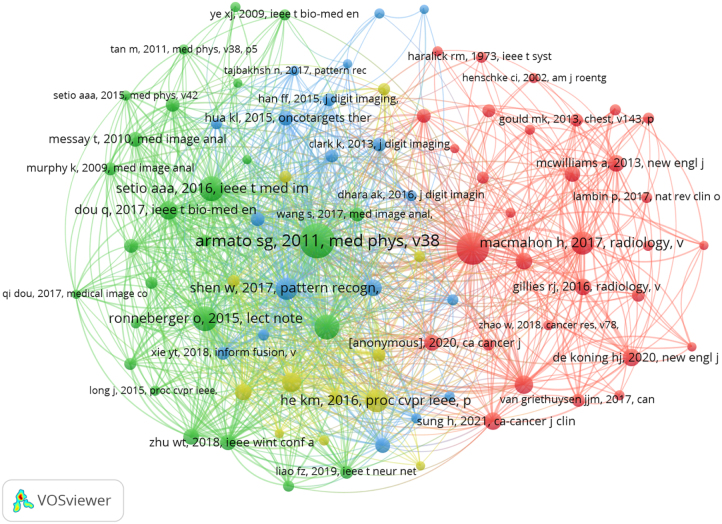
The visualization of cocited references in the field of artificial intelligence and lung nodule research.

Table 3 delineates the 10 most-cited publications in lung nodules AI research. The seminal reference is Armato’s 2011 Medical Physics study (citation count: 506), which established the first large-scale, publicly accessible international database of thoracic CT imaging for lung cancer research. This landmark resource specifically curates “gold standard” annotations for pulmonary nodules, including precise spatial localization, boundary delineation, volumetric measurements, and morphological characteristics, providing an indispensable benchmark for developing, validating, and comparing computer-aided detection/diagnosis (CAD) algorithms. As a foundational resource, this dataset has significantly catalyzed advances in oncological imaging AI research and remains a critical tool for scientific discovery in the field^[69]^. Clustering cocited references helps to uncover the frontiers of this domain (Fig. 8). The positioning of nodes within these clusters identifies deep CNNs, medical image segmentation of lung nodules, and benign-malignant prediction as the key frontiers in AI-driven lung nodules research.

**Table 3:** Top 10 cocited references in the field of artificial intelligence and lung nodule research.

Rank	Title	Year, journal	First author	Total citations	DOI
1	The Lung Image Database Consortium (LIDC) and Image Database Resource Initiative (IDRI): a completed reference database of lung nodules on CT scans	2011, Medical physics	Samuel G. Armato III	506	10.1118/1.3528204
2	Reduced lung-cancer mortality with low-dose computed tomographic screening	2011, The new England journal of medicine	National Lung Screening Trial Research Team	438	10.1056/nejmoa1102873
3	U-Net: Convolutional networks for biomedical image segmentation	2015, Medical Image Computing and Computer-Assisted Intervention – MICCAI 2015	Olaf Ronneberger	281	10.1007/978-3-319-24 574-4_28
4	Validation, comparison, and combination of algorithms for automatic detection of pulmonary nodules in computed tomography images: the LUNA16 challenge	2017, Medical image analysis	Arnaud Arindra Adiyoso Setio	272	10.1016/j.media.2017.06.015
5	Pulmonary nodule detection in CT images: false positive reduction using multiview convolutional networks	2016, IEEE transactions on medical imaging	Arnaud Arindra Adiyoso Setio	264	10.1109/tmi.2016.2536809
6	Deep residual learning for image recognition	2016, IEEE	Kaiming He	243	10.1109/cvpr.2016.90
7	Guidelines for management of incidental pulmonary nodules detected on CT images: from the Fleischner Society 2017	2017, Radiology	Heber MacMahon	232	10.1148/radiol.2017161659
8	Multicrop convolutional neural networks for lung nodule malignancy suspiciousness classification	2017, Pattern Recognition	Wei Shen	216	10.1016/j.patcog.2016.05.029
9	ImageNet classification with deep convolutional neural networks	2017, Communications of the ACM	Alex Krizhevsky	176	10.1145/3 065 386
10	End-to-end lung cancer screening with three-dimensional deep learning on low-dose chest computed tomography	2019, Nature Medicine	Diego Ardila	164	10.1038/s41591-019-0447-x

#### Analysis of citation bursts in references

**Figure 9.**
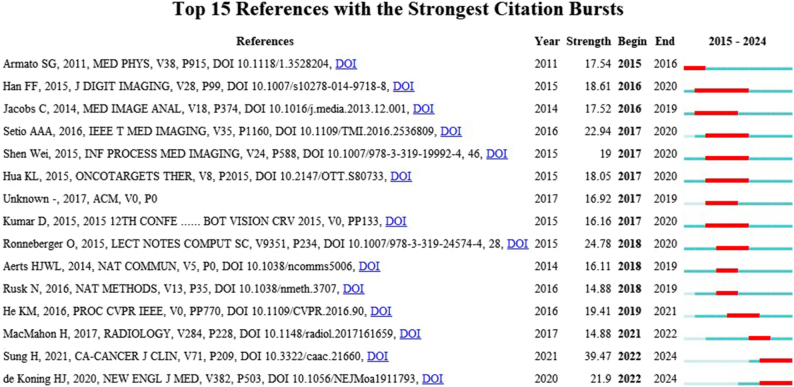
Top 15 references with strong citation bursts. A red bar indicates high citations in that year.

Figure 9 identifies the 15 references with the strongest citation bursts. Chronological citation patterns (2015–2024) are denoted by blue bands, while red segments mark burst periods (minimum duration: 1 year). The most prominent citation burst (intensity: 39.47, 2022–2024) corresponds to Sung *et al*^[70]^’s landmark study “Global Cancer Statistics 2020: GLOBOCAN Estimates of Incidence and Mortality Worldwide for 36 Cancers in 185 Countries.” This foundational analysis comprehensively quantified global cancer epidemiology, delineating incidence/mortality patterns for 36 malignancies across 185 countries. By mapping evolving oncological disease burdens, it established critical epidemiological baselines instrumental for global cancer control initiatives^[70]^. The second strongest citation burst (intensity: 24.78, 2018–2020) corresponds to Ronneberger *et al*^[71]^’s seminal work “U-Net: Convolutional Networks for Biomedical Image Segmentation.” This breakthrough introduced a symmetric encoder-decoder architecture that achieves subpixel segmentation accuracy under scarce annotation constraints. The framework specifically addresses the critical challenge of delineating morphologically variable small-scale anatomical targets with ambiguous boundaries in medical imaging. It has fundamentally reshaped computational approaches to lesion detection, surgical navigation, and quantitative pathology analysis^[71]^.

### Keywords analysis

#### Cooccurrence analysis

**Figure 10.**
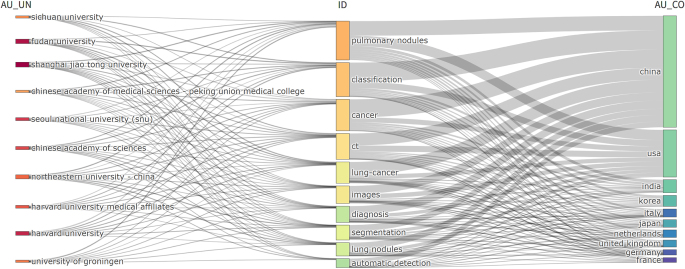
Network visualization between the top countries, organizations and keywords.

The institution-country thematic association network (Fig. 10) delineates global collaborative patterns and intellectual focal points in AI-driven lung nodules research. Institutional research priorities demonstrate distinct specialization: Shanghai Jiao Tong University leads in “classification,” “lung cancer diagnostics,” and “CT imaging” domains, whereas Harvard University concentrates on “segmentation algorithms” and “diagnostic system optimization.” At the national level, China and the United States collectively account for over 95% of high-frequency thematic keywords, establishing a dual-pole monopoly over core research domains.

**Figure 11.**
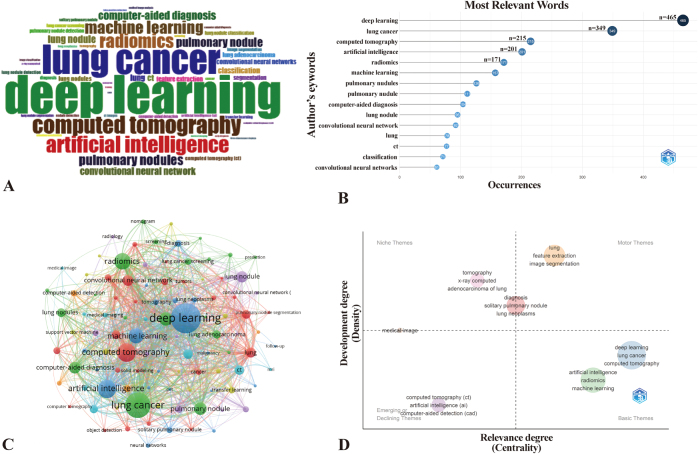
Keyword analysis and visualization: (A) high-frequency keyword word cloud in the field of lung nodules and artificial intelligence; (B) the 15 most commonly used thematic keywords in the field of lung nodules and artificial intelligence; (C) keyword visualization presentation in the field of lung nodules and artificial intelligence; (D) thematic map visualization presentation in the field of lung nodules and artificial intelligence.

Bibliometric analysis of 2933 keywords reveals the evolving intellectual architecture of AI-enabled lung nodules research. High-frequency themes dominate the knowledge landscape: deep learning (477 occurrences), lung cancer (356 occurrences), CT imaging (216 occurrences), AI (204 occurrences), radiomics (173 occurrences), and machine learning (157 occurrences) form the foundational framework visualized in Figure 11A–B. Figure 11C delineates cooccurrence patterns among 104 keywords (appearing ≥10 times) through three-dimensional encoding principles: nodal size corresponds to frequency, chromatic classification indicates cluster affiliation, and inter-node distance reflects conceptual correlation strength – organized within seven distinct thematic clusters. Temporal evolution trajectories emerge in Supplemental Digital Content Supplementary Figure 2 available at: http://links.lww.com/JS9/G526, where chromatic gradients map research emphasis transitions: deep-to-light blue hues signify early-phase concentration on computer-aided diagnosis (CAD), pulmonary nodules, and lesion classification (2009–2016), while light-to-deep yellow gradations mark contemporary prioritization of three-dimensional reconstruction, attention-driven neural networks, and clinical nomogram development (2021–2024). Strategic coordinate analysis (Fig. 11D) positions research domains across centrality-density vectors, identifying feature extraction, segmentation algorithms, deep learning, and machine learning in the upper-right quadrant (active themes with paradigm-defining importance), whereas computer-aided detection resides in the upper-left quadrant (technically mature but diminishing-impact niche domains). This cartographic synthesis confirms the progressive migration from traditional CAD frameworks toward adaptive deep neural architectures.

#### Keywords with citation outbreaks

**Figure 12.**
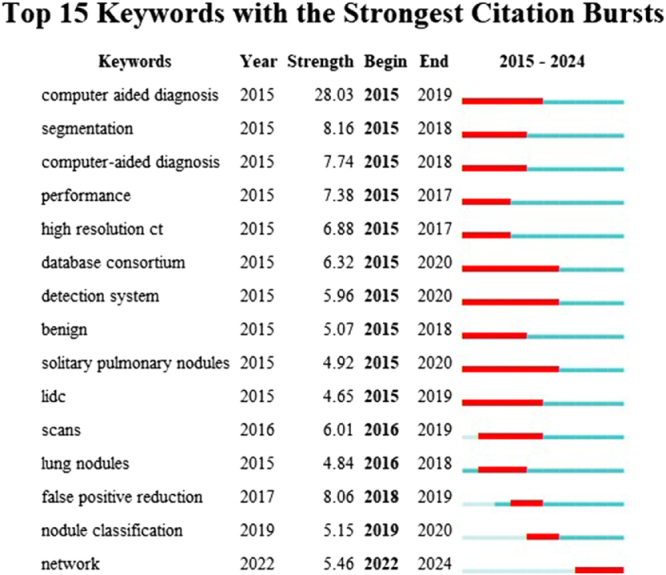
Top 15 keywords with strong citation bursts in the field of artificial intelligence and lung nodule research.

Citation burst analysis reveals evolving research priorities by identifying keywords demonstrating intense scholarly engagement across specific temporal intervals. Figure 12 illustrates the top 15 keywords with the strongest citation bursts from 2015–2024, dominated by three high-intensity classifications: CAD (strength = 28.03), segmentation techniques (strength = 8.16), and false-positive reduction (strength = 8.06). Particularly noteworthy is “deep learning networks,” exhibiting a remarkable surge period (strength = 5.46, 2022–2024) that positions it as an emergent research frontier. These burst patterns collectively identify high-impact trajectories currently shaping scholarly discourse and likely to define near-term investigative priorities in AI-enabled lung nodules research.

## Discussion

### Global trends in the investigation of pulmonary nodules

This bibliometric study systematically analyzed 1779 publications focusing on AI applications in pulmonary nodules research between January 2015 and December 2024. The analysis reveals consistent expansion in academic output: annual publications increased from 32 articles during the initial phase of disciplinary establishment in 2015 to a period of accelerated growth by 2024. Notably, the majority of cumulative research output was produced during 2020–2024, peaking in 2023. This developmental trajectory was primarily driven by three key technological catalysts: (1) enhanced computational capacity and optimized algorithm architectures; (2) establishment of large-scale imaging databases; and (3) the sharp increase in nodule detection demand driven by the widespread adoption of low-dose CT screening in the postpandemic era – collectively highlighting the clinical urgency in pulmonary nodule management^[47,72]^. Given the pulmonary nodules’ critical role in early oncological interception and substantial heterogeneity, this domain has rapidly evolved into a research priority. Consequently, many oncologists and pulmonologists strategically redirected efforts toward AI-nodule applications, fundamentally reorienting research paradigms. Recent evidence, however, indicates that such pandemic-driven research pivoting incurred significant “academic transition penalties”: Scholars deviating from core expertise to emergent domains generated work with disproportionately reduced scientific impact, aligning with this field’s recent suboptimal citation performance^[73,74]^. Nevertheless, the sustained growth in publications highlights the transformative impact of AI, particularly deep learning and computer vision systems, in revolutionizing the management of pulmonary nodules. These technologies have been shown to enhance detection sensitivity, refine malignancy risk stratification, and guide therapeutic decision-making. Post-2023 citation patterns notably suggest an accelerating transition from foundational algorithm development to clinical deployment, where AI-powered diagnostic frameworks are optimizing core clinical pathways such as nodule identification, risk quantification, and intervention planning. This trajectory reflects the progressive assimilation of the field into routine medical practice, signaling an imminent paradigm shift toward AI-integrated thoracic oncology workflows.

Author analysis indicates that 2642 authors contributed to the 1779 publications, with high-output author analysis showing that Colin Jacobs and Jin Mo Goo are the most representative researchers in this field. Colin Jacobs’ team focuses on algorithms for assessing the malignant risk of pulmonary nodules. Their 2021 study, published in Radiology, developed a deep learning algorithm for evaluating the malignant risk of pulmonary nodules detected during CT screening, demonstrating excellent performance in clinical practice^[75]^. Clinical translation of AI is prioritized in investigations conducted by Goo, JM’s research team. A multicenter randomized controlled trial involving 10 476 participants in 2023 demonstrated that AI-assisted screening significantly improved the detection rate of pulmonary nodules compared with conventional screening methods (0.59% vs. 0.25%)^[76]^. Among cocited authors, S.G. Armato (citation count = 765) is recognized as the foremost authority. The seminal 2011 publication “The Lung Image Database Consortium (LIDC) and Image Database Resource Initiative (IDRI): A Completed Reference Database of Lung Nodules on CT Scans” remains the most-cited work^[69]^. This foundational study established a publicly accessible benchmark dataset that enables standardized development, training, validation, and comparative assessment of CAD algorithms. The dataset’s implementation is documented to have substantially accelerated AI-driven pulmonary nodule imaging research, and it continues to be utilized as an essential methodological resource within the discipline. Author collaboration network analysis reveals that research communities exhibit distinct clustered patterns at institutional and regional levels, with strong and stable cooperative relationships established within individual clusters. However, collaborations across teams, institutions, and regions remain relatively limited, which to some extent restricts knowledge flow and methodological innovation. Promoting cross-team collaboration has been recognized as a key strategy to drive breakthrough progress in this field. By actively integrating diverse research perspectives and advanced methodologies, optimizing resource allocation, and establishing collaborative research mechanisms, it is expected to break down academic barriers, stimulate innovation, and foster more innovative and influential research outcomes. Furthermore, encouraging open science practices and data sharing will further contribute to the formation of a high-quality collaborative ecosystem.

AI research in pulmonary nodules demonstrates significant geographical disparities, with distinct developmental trajectories and academic focus observed across different regions. The United States maintains a leading position in academic influence, as reflected in its globally highest citation frequency and consistent output of high-impact publications. However, the academic focus in this field has notably shifted eastward in recent years, with East Asia – particularly China – emerging as the core of research activity. Shanghai Jiao Tong University and Fudan University in China are at the international forefront of this domain, with their research primarily covering key areas such as clinical diagnosis and treatment of pulmonary nodules, discrimination between benign and malignant lesions, and risk prediction. For example, Fudan University has achieved a major breakthrough in clinical research by proposing the concept of the “surgical cure window” – a specific clinical or pathological stage during which surgical resection achieves a 100% 5-year survival rate, independent of the exact timing of intervention^[77]^. This concept provides critical support for clinical decision-making and underscores the clinical value of active surveillance as an effective management strategy. Shanghai Jiao Tong University systematically analyzed the DNA methylation profiles of benign and malignant pulmonary nodules, identifying characteristic methylation regions that can distinguish between the two types of lesions. Based on these findings, the university developed a novel diagnostic tool named LUNG-TRAC. Multicenter validation demonstrated that the model achieves an AUC of 0.761 for predicting benign and malignant pulmonary nodules, with a specificity of 80.8%, effectively reducing misdiagnosis and unnecessary surgeries^[78]^. The emergence of East Asia as a key region in this field is driven by a combination of factors: sustained government funding, the integration of lung cancer screening into national public health programs, the establishment of well-coordinated research alliances, and a unique epidemiological profile – particularly the high incidence of GGNs associated with lung adenocarcinoma among nonsmoking Asian women. Notably, studies have confirmed that traditional risk prediction models developed primarily based on Caucasian populations show significantly reduced applicability in these specific demographic groups, revealing limitations in the cross-ethnic generalizability of existing models^[79]^. Moreover, the accelerating demographic aging in East Asia introduces unique challenges in pulmonary nodule management for elderly patients: Existing technologies inadequately predict disease progression trajectories in this population, leading to premature resection of indolent nodules and unnecessary surgical risks with associated complications. Concurrently, prolonged indolent phases, slow progression rates, and limited life-expectancy impact in elderly patients render fixed-interval surveillance protocols suboptimal for balancing overdiagnosis against delayed detection dilemmas. These complexities fundamentally increase the difficulty of nodule characterization and risk stratification, thereby intensifying research focus on pulmonary nodules. AI has consequently emerged as a pivotal methodological tool, driving heterogeneous publication outputs that reflect profound regional differences in scientific priorities. This paradigm explains both the proliferation of nodule phenotype studies targeting East Asian populations – contributing substantially to regional publication volume – and the critical necessity of incorporating population-specific variables when developing clinical guidelines and refining risk assessment models. AI demonstrates particular value in addressing these challenges. Through advanced data pattern recognition and machine learning capabilities, AI-powered risk stratification significantly outperforms conventional approaches in diagnostic accuracy for nonsmoking women and elderly populations. This technology provides crucial support for early detection and treatment decisions, potentially reducing disease-specific mortality in these demographics. Consequently, advancing AI development and clinical implementation represents an imperative frontier in respiratory medicine.

In terms of journals, highly cited research outcomes are primarily concentrated in internationally authoritative journals in the field of radiology (such as Radiology) and top medical imaging journals (such as IEEE Transactions on Medical Imaging), while top conference proceedings in the field of computer science also contribute significantly, forming a complementary cross-disciplinary pattern. This further indicates that AI-driven nodule detection, classification, and prognostic prediction require deep integration of medical imaging analysis and deep learning algorithms. The resulting interdisciplinary research ecosystem promotes the simultaneous emergence of core outcomes in professional journals and top conferences.

Keyword analysis has clearly revealed research hotspots and developmental trajectories in the field of AI for pulmonary nodules. Within this domain, “deep learning” and “machine learning” together form a dual methodological core, exhibiting significant differences in both technical approaches and clinical applications. At the model construction level, deep learning – particularly CNNs and U-Net architectures – is primarily applied to end-to-end analysis of medical images. Its core advantages lie in automatic feature extraction and precise semantic segmentation capabilities, especially in identifying subtle nodule characteristics. Breakthroughs in technologies such as CNNs, exemplified by the exceptional performance of AlexNet in the 2012 ImageNet competition, marked the beginning of a new era in medical image analysis. Compared to traditional CAD systems, CNNs significantly enhance the sensitivity and efficacy of pulmonary nodule diagnosis by autonomously learning high-level image features^[80]^. This technology enables dynamic quantification of tumor volume changes, synchronous tracking of multiple lesions, genotypic association analysis of intratumoral heterogeneity, and prediction of clinical outcomes through intelligent comparison of individual cases with large-scale databases^[81]^. Furthermore, the strong generalization capability demonstrated by deep learning models across multimodal imaging data provides substantial support for their application in diverse clinical scenarios^[82]^. However, such methods impose substantial demands on data volume and computational resources, and their decision-making processes continue to face interpretability limitations. Further advancements are still required, including more efficient network architectures, more robust self-supervised approaches, and standardized validation through multicenter, large-scale studies^[83]^. In contrast, traditional machine learning methods – such as support vector machines (SVM) and random forests – rely more on handcrafted radiomic features. They demonstrate better stability under limited data conditions and offer superior clinical interpretability. For example, a retrospective study involving 3355 patients employed a five-fold cross-validation strategy to systematically compare the diagnostic performance of multiple models. Results indicated that the attention-based feedforward neural network (Atten_FNN) achieved the best performance with an AUC of 0.82, accuracy of 0.75, sensitivity of 0.77, and an F1-score of 0.80. Interpretability analysis using the SHAP framework further identified key predictive factors, including demographic characteristics (age, sex, BMI), CT imaging parameters (maximum nodule diameter, morphological features, density, degree of calcification, and presence of ground-glass opacity), and laboratory biomarkers (neuroendocrine markers and carcinoembryonic antigen levels)^[84]^.

Through keyword cooccurrence and cluster analysis, we observed that the two methodologies have formed distinct research directions: deep learning is primarily focused on semantic segmentation and three-dimensional reconstruction of CT images, while machine learning is more dedicated to classification prediction based on clinical data and imaging features. This coexistence reflects the multidimensional development of AI research in pulmonary nodules – deep learning demonstrates exceptional performance in mining complex features, whereas machine learning maintains a vital role in clinical decision support due to its practicality and interpretability. Together, they constitute the methodological framework of this field.

Keyword analysis reveals a distinct clinical application orientation in AI research for pulmonary nodules. High-frequency terms such as “lung cancer” and “CT” constitute the core research focus in this field, highlighting the academic commitment to advancing early lung cancer screening and prevention through CT imaging technology. Meanwhile, the emergence of emerging keywords like “computer-aided diagnosis” and “false-positive rate optimization” reflects the systematic integration of AI technologies into clinical workflows. Studies indicate that a high false-positive rate remains a major challenge in the current application of AI for pulmonary nodule identification and diagnosis. In recent years, quantitative biomarker models based on multimodal imaging phenomics have significantly improved the discrimination between benign and malignant pulmonary nodules while effectively reducing false-positive rates in CT screening. However, due to the high heterogeneity of pulmonary nodules and the prevalent issue of class imbalance in training data, the generalization capability of these models remains constrained^[24]^. Therefore, further breakthroughs are still needed in model generalization performance, multicenter clinical validation, and interpretability of results to better meet the demands of real-world clinical applications.

The frequent emergence of “network” keywords in recent years signifies that pulmonary nodule research has entered a new phase centered on deep learning, with CNNs and GANs being particularly widely applied. In CT image analysis of pulmonary nodules, CNNs have become one of the core technologies. They can automatically learn the morphology, size, density, and edge characteristics of pulmonary nodules, enabling high-precision detection and segmentation. Leveraging transfer learning strategies, CNNs demonstrate excellent generalization capability even with limited medical data, and are extensively used in tasks such as distinguishing benign from malignant nodules, risk stratification, and growth trend prediction. For example, models based on the U-Net architecture achieve pixel-level segmentation, while networks like ResNet and Inception significantly improve accuracy and specificity in classification tasks^[85,86]^. Through end-to-end learning mechanisms, CNNs effectively reduce reliance on manual feature design, providing scalable and robust solutions for intelligent diagnosis of pulmonary nodules.

GANs are a deep learning framework based on game theory, achieving collaborative optimization through adversarial training between a generator and a discriminator. In pulmonary nodule research, GANs are primarily employed to address the scarcity of medical imaging data and class imbalance issues. This technology can generate synthetic pulmonary nodule images with diverse pathological semantics, effectively expanding the training dataset and significantly enhancing the model’s generalization capability and robustness^[87]^. Furthermore, conditional generative adversarial networks (cGANs) enable super-resolution reconstruction and multimodal fusion of CT images, improving the imaging quality of small nodules and providing technical support for cross-modal diagnosis^[88,89]^. In terms of data augmentation, three-dimensional conditional generative adversarial networks (3D cGANs) can generate realistic and diverse synthetic medical images, effectively supplementing scarce category samples^[90]^. Additionally, the combination of physics-based data augmentation methods and GANs further enhances the model’s generalization capability and robustness in practical applications^[91,92]^. These technologies collectively establish a new research paradigm that integrates data-driven and model optimization approaches, demonstrating significant value in improving image quality, expanding datasets, and enhancing model stability^[93]^.

### Research challenges and outlook of AI in lung nodules

AI technology demonstrates substantial potential for the precise diagnosis of pulmonary nodules and informed treatment decision-making. With ongoing advancements in algorithm optimization and multimodal data fusion techniques, AI is expected to play an increasingly critical role in key areas such as early screening of pulmonary nodules, development of personalized treatment plans, and dynamic prognostic evaluation. However, current AI-based pulmonary nodule management models have yet to be widely adopted in clinical practice, and a significant gap remains between the performance reported in research settings and real-world clinical application. Furthermore, the acceptance of AI among both medical professionals and patients remains low. A 2023 multinational survey encompassing 13 806 patients across 43 countries revealed that although overall perceptions of AI were slightly more positive than negative, only 4.4% of patients were willing to allow AI to fully replace physicians, with the vast majority preferring AI to serve in an assistive role^[94]^. Thus, the application of AI in the field of pulmonary nodules still faces several critical challenges and research problems that require urgent resolution.

#### Data heterogeneity and model generalization limitations

Data heterogeneity represents a critical constraint on the generalizability of AI models for pulmonary nodule analysis, primarily due to substantial selection bias inherent in the retrospective data used for model training. Such heterogeneity manifests in two key aspects: diversity in patient populations and variations in imaging equipment and protocols, which collectively hinder the models’ ability to fully capture the complexity of real-world clinical settings, thereby limiting their broad applicability. Retrospective data often lack adequate representation across different age groups, genders, ethnicities, and underlying disease distributions, resulting in degraded diagnostic performance of trained models when applied to specific subpopulations. For instance, models trained predominantly on Western populations generally exhibit lower accuracy in assessing the malignancy risk of pulmonary nodules in Asian cohorts^[24]^. Moreover, geographic concentrations and demographic biases in training data can introduce diagnostic preferences, further compromising model performance across diverse patient subgroups.

Technical variations in imaging equipment represent a critical factor constraining the generalizability of AI models. Differences in CT scanner models, scanning protocols, and reconstruction kernels significantly alter the physical characteristics of images, inducing variations in radiomic features that consequently degrade model detection performance. For instance, when the slice thickness increases from 1 mm to 5 mm, the detection performance of AI-assisted tools for pulmonary nodules may decrease by 49.3%^[95]^. Additionally, processing the same nodule with different reconstruction parameters can lead to substantial differences in feature values; such changes are sufficient to render models trained on specific parameters ineffective when applied to data from different equipment sources^[96]^. Furthermore, studies indicate that low-dose CT, due to higher image noise, reduces the detection sensitivity of AI for small nodules (diameter <5 mm) by 10.1%, further highlighting the necessity of technical standardization in imaging AI applications^[97]^.

The limitations of retrospective data create a negative feedback loop for model generalizability. On one hand, such data fail to comprehensively capture the diversity and complexity of real-world clinical scenarios; on the other hand, models trained on this data often exhibit significant performance degradation when deployed in new healthcare settings or different patient populations due to variations in equipment parameters and patient characteristics. This vicious cycle constrains the reliable clinical application of AI, and despite demonstrating certain potential in screening contexts, its practical deployment still requires rigorous professional supervision. Current research indicates that AI-assisted CT image analysis based on retrospective data generally carries a high risk of bias and lacks sufficient evidence for improving clinical outcomes or cost-effectiveness ^[98,99]^.

To systematically address the aforementioned challenges, it is imperative to establish a prospective, multicenter collaborative framework for data acquisition and quality control, with emphasis on both patient population diversity and imaging equipment standardization. During the data acquisition phase, strict adherence to the Image Biomarker Standardization Initiative (IBSI) guidelines is recommended^[100]^. This includes standardizing resampling parameters during image preprocessing (recommended voxel size: 1 × 1 × 1 mm^3^) and establishing multicenter consensus on scanning protocols. Specific measures should involve fixed tube voltage (e.g., 120 kVp), automatic tube current modulation (effective mAs range: 30–50), and thin-slice scanning (slice thickness ≤ 1 mm)^[24,101]^. In the model development phase, heterogeneous data augmentation strategies should be introduced to actively generate simulated data encompassing various scanning parameter combinations. Federated learning techniques can be employed to achieve cross-institutional domain adaptation without centralizing sensitive data. At the clinical implementation level, systematic training for radiologists should be enhanced following the Lung-RADS standard to improve interpretation consistency. Concurrently, automated quality control tools should be developed to quantitatively evaluate and screen key metrics, including image noise, spatial resolution, and CT number accuracy.

By optimizing data acquisition workflows, integrating transfer learning techniques to enhance model adaptability across devices and populations, and conducting large-scale, multicenter external validations using heterogeneous datasets, the robustness and generalizability of AI models in real-world scanning environments can be significantly improved. These advances will facilitate the substantial transition of intelligent pulmonary nodule analysis from experimental research to scalable clinical applications.

#### Doctor-patient relationship and trust issues in AI-assisted decision making

The rapid advancement of medical AI faces a critical challenge due to the inherent lack of transparency in “black-box” algorithms. In the diagnosis and clinical risk stratification of pulmonary nodules, the complexity of AI decision-making processes results in poor interpretability, making it difficult for clinicians to trace and validate the underlying rationale. This diminishes trust and willingness to adopt these tools in clinical practice. Insufficient model transparency may not only compromise the quality of diagnostic and therapeutic decisions but also further impair treatment outcomes^[102–104]^. Patients with pulmonary nodules often experience significant cancer-related anxiety. If clinicians are unable to explain the decision-making logic of AI-assisted diagnostics, they will face substantial challenges in conveying diagnostic findings to patients. Such communication barriers may exacerbate patients’ anxiety and depressive symptoms, ultimately exerting negative impacts on clinical outcomes^[105]^.

Furthermore, clinicians’ understanding and proficiency with AI represent a significant human-factor barrier to its adoption. AI systems typically provide probabilistic assessments of nodule malignancy rather than deterministic diagnoses, requiring physicians to develop new interpretive and judgment competencies. Currently, most clinicians lack systematic training in AI working principles, limitations, and interpretation methods, which frequently leads to two problematic extremes: either over-relying on AI outputs and compromising clinical autonomy, or completely dismissing its valid recommendations, resulting in technological underutilization. Therefore, it is crucial to not only provide operational training but also foster a paradigm shift in clinical decision-making approaches, while establishing long-term training mechanisms that incorporate output verification and ongoing feedback systems.

In response to the aforementioned challenges, research focus has gradually shifted toward the development of explainable artificial intelligence (XAI). XAI aims to enhance user trust by revealing the decision-making mechanisms of models, employing methods such as generating visual explanations and constructing interpretable surrogate models for comparative analysis. In pulmonary nodule diagnosis, one study improved model interpretability by decomposing time-enhancement curves into three physiological dimensions – blood flow, permeability, and metabolic activity – extracting radiomic features such as peak enhancement values and perfusion slopes to construct an interpretable classification framework. Using machine learning algorithms, the diagnostic weight of each parameter was quantified^[106]^.

In terms of building physician–patient trust, clinicians should demonstrate “rational transparency” during communication by clearly explaining the role and limitations of AI tools. This helps patients understand their supplementary rather than substitutive function, thereby enhancing a sense of security and trust^[107]^. In shared decision-making, physicians must fully respect patients’ opinions and emotional needs, ensuring they have a voice in the process to improve recognition of and adherence to treatment plans^[108,109]^.

Against this backdrop, targeted education and training for clinicians are particularly crucial. Training should cover interpreting AI decisions, patient communication strategies, and maintaining humanistic care and empathic capacities with technological assistance. Through systematic training, physicians can both effectively utilize AI to improve diagnostic and therapeutic proficiency and maintain and strengthen patient trust.

#### Regulatory oversight and ethical issues

The rapid integration and deepening application of AI in healthcare have brought to the forefront critical bottlenecks in its development, including the absence of regulatory frameworks, data ethics challenges, and algorithmic fairness concerns. Currently, most countries worldwide lack specialized regulatory systems for medical AI, resulting in ambiguous accountability in clinical practice. As the ultimate decision-makers in clinical settings, physicians who fail to adequately review and assess the risks associated with AI-generated outputs may face potential legal liabilities^[110]^. Simultaneously, the large-scale health data utilized by AI models involve highly sensitive personal information. Growing public expectations regarding informed consent and data anonymization mean that improper data handling not only undermines trust between patients and healthcare providers but may also lead to compliance disputes. Furthermore, a lack of diversity in training data may exacerbate health service disparities, limiting access to accurate diagnosis and treatment for specific demographic groups^[111]^.

To systematically address these challenges, it is imperative to establish specialized regulatory frameworks that clarify accountability and responsibility. A robust medical data governance system aligned with international standards such as the General Data Protection Regulation (GDPR) should be implemented, while multicenter collaborative mechanisms should be enhanced to improve the representativeness and coverage of data samples. Concurrently, healthcare professionals require strengthened training in both the application of AI tools and ethical assessment. By ensuring data security and algorithmic fairness, these measures will promote the standardized and sustainable development of medical AI.

#### Barriers to AI integration in clinical practice

The insufficient integration between AI tools for pulmonary nodules and existing hospital Picture Archiving and Communication Systems (PACS) has become a critical barrier to their widespread clinical adoption. Although significant progress has been made in AI and large-scale model technologies in recent years, leading to a growing number of medical AI applications for pulmonary nodule analysis, most conventional PACS – constrained by outdated architecture and upgrade challenges – struggle to effectively support increasingly sophisticated and diverse AI functionalities. The current clinical diagnostic workflow heavily relies on PACS for image retrieval, processing, and reporting. However, many existing AI-based pulmonary nodule systems operate on standalone platforms, resulting in “system isolation.” This fragmented environment forces physicians to frequently switch between different interfaces and operational workflows, which not only reduces diagnostic efficiency but also causes workflow interruptions and loss of productivity^[112]^.

To achieve seamless integration of AI systems into clinical workflows, a systematic engineering strategy is essential. The primary objective is to position AI as an efficient and seamlessly embedded auxiliary tool for physicians rather than a standalone module that adds operational complexity. At the technical level, systems should comply with international standards such as DICOM to enable interoperable data exchange among PACS, RIS, and Hospital Information Systems (HIS). Through embedded integration or middleware-based cloud service models, AI functionalities can be effortlessly incorporated into physicians’ daily diagnostic workstations. This allows automatic image acquisition, intelligent analysis, and structured reporting of results, thereby eliminating efficiency losses due to cross-platform operations^[34]^. A notable example is United Imaging Intelligence’s next-generation AI PACS platform, which incorporates innovative architectural designs to build an integrated imaging platform based on a digital-intelligent infrastructure, serving as a unified multimodal imaging data hub. Such platforms centralize and modularize AI capabilities. The structured reports generated can be automatically transmitted to the RIS and embedded into hospital-customized report templates. Physicians need only modify and confirm key findings, significantly streamlining reporting processes and enhancing workflow continuity and efficiency.

#### Lack of payment mechanism

The absence of a well-defined reimbursement mechanism has become a critical economic barrier hindering the clinical application and widespread adoption of AI technology for pulmonary nodule diagnosis. Studies have shown that the use of AI can achieve a minimum net cost saving of approximately $72 per patient undergoing pulmonary nodule screening^[26]^. However, most current AI-assisted diagnostic technologies for pulmonary nodules have not yet established independent billing systems, posing significant challenges within existing medical payment frameworks. In current healthcare systems, reimbursement is primarily based on predefined diagnostic and treatment items covered by medical insurance payers. Since AI-assisted diagnosis has not been widely included in medical insurance reimbursement catalogs, healthcare institutions demonstrate significantly reduced procurement motivation, resulting in a “disconnect between technological application and clinical translation.” This issue is particularly evident in fee-for-service systems, where the lack of independent billing codes and standardized reimbursement pathways for innovative services such as “AI-assisted image interpretation” makes it difficult for medical institutions to accurately calculate and demonstrate return on investment. This severely constrains the sustainable development of the technology’s business model.

To overcome this bottleneck, there is an urgent need to validate the clinical and operational benefits of AI technology, such as improving diagnostic accuracy, reducing reporting time, and optimizing resource allocation, using real-world research evidence. Simultaneously, it is essential to actively explore diversified payment mechanisms aligned with value-based healthcare principles, including applying for new medical insurance billing items, establishing a tiered pricing system based on clinical value, or developing innovative profit-sharing models that distribute value gains with hospitals. Only by addressing these commercialization challenges can AI technology for pulmonary nodules successfully transition from technological innovation to large-scale clinical application.

## Advantages and limitations

This study is the first to employ bibliometric methods to systematically review the development of AI in the interdisciplinary field of pulmonary nodule research from 2014 to 2024. It integrates 1779 original research articles and review papers, comprehensively revealing the research evolution path, distribution of hotspot topics, and collaboration network patterns within the field, thereby providing a decision-making basis for future academic planning and clinical translation strategies.

However, this study has several limitations, with the primary and most significant being the selection bias resulting from the use of a single data source. The literature retrieval was conducted solely using the WoS Core Collection. Although this database includes high-quality, high-impact peer-reviewed journals from around the world and is widely regarded as an authoritative source in bibliometric research, the exclusion of other important sources such as PubMed, Scopus, Embase, and other specialized databases constitutes a major methodological limitation that needs to be addressed in future bibliometric studies. Since WoS tends to prioritize English-language and high-impact journals from Europe and North America, this analysis may systematically underestimate high-quality research published in non-English journals, regionally important publications, or specialized databases. This could affect the comprehensiveness and representativeness of the research overview and may lead to biases in hotspot identification and trend interpretation. To address this issue, future bibliometric analyses should incorporate multilingual databases and conduct stratified analyses by research type to more accurately distinguish between early-stage research and clinically applied technologies. Second, this study focuses on the application of AI in the diagnosis of pulmonary nodules, a field characterized by extremely rapid technological iteration. Although every effort was made to include the most recent available literature, the inherent time lag in retrieval, inclusion, and citation analysis means that some recently published breakthrough studies and the latest technological advances may not have been fully captured or reflected. Therefore, ongoing updates and tracking analyses are necessary in the future. Additionally, bibliometric methods themselves have inherent limitations, such as citation preferences, self-citation practices, and differences in citation norms across disciplines. These factors may, to some extent, affect the objectivity of assessments regarding the influence of authors, institutions, and countries/regions. Furthermore, this study relied on a predefined keyword strategy for literature retrieval. Despite multiple optimizations, discrepancies in terminology usage or inconsistent indexing may still have led to the omission of some relevant literature.

Despite the above limitations, this study, through a systematic analysis of the high-quality literature included in the WoS Core Collection, still provides a relatively clear outline of the knowledge structure, research hotspots, and development trajectory of the field, offering valuable reference for researchers to grasp the overall discipline and its evolutionary dynamics.

## Conclusion

This study provides a systematic review of the research progress on AI applied to pulmonary nodule management from 2015 to 2024. It summarizes its practical applications in nodule detection, benign-malignant differentiation, invasiveness prediction, and overall management throughout the entire patient pathway – from preoperative planning and intraoperative guidance to postoperative follow-up. Furthermore, the review outlines future development directions, highlights clinical research priorities, analyzes differences in translational pathways, and identifies unique challenges in the field, thereby enhancing the utility of these research outcomes for clinical practice.

Although AI demonstrates considerable potential in the diagnosis and treatment of pulmonary nodules, its widespread clinical adoption remains hindered by several challenges. These include limited model generalizability, a lack of trust among physicians and patients, incomplete regulatory and ethical frameworks, difficulties in integrating AI into clinical workflows, and the absence of reimbursement mechanisms. To address these barriers, future efforts should focus on fostering interdisciplinary collaboration, promoting standardized data collection, developing interpretable AI algorithms, improving regulatory systems, and innovating payment models. Such strategies are essential to promote the transition of AI from experimental research to real-world clinical practice, ultimately advancing precision medicine in early diagnosis and treatment and improving patient outcomes.

## Data Availability

The datasets analyzed during the current study are available from the corresponding author on reasonable request.

## Research registration unique identifying number (UIN)

1. Name of the registry: not required. 2. Unique identifying number or registration ID: not required. 3. Hyperlink to your specific registration (must be publicly accessible and checked): not required.
